# Limited effects of early snowmelt on plants, decomposers, and soil nutrients in Arctic tundra soils

**DOI:** 10.1002/ece3.4870

**Published:** 2019-01-24

**Authors:** Anthony Darrouzet‐Nardi, Heidi Steltzer, Patrick F. Sullivan, Aliza Segal, Amanda M. Koltz, Carolyn Livensperger, Joshua P. Schimel, Michael N. Weintraub

**Affiliations:** ^1^ Department of Biological Sciences University of Texas at El Paso El Paso Texas; ^2^ Department of Environmental Sciences University of Toledo Toledo Ohio; ^3^ Biology Department Fort Lewis College Durango Colorado; ^4^ Environment and Natural Resources Institute University of Alaska Anchorage Anchorage Alaska; ^5^ Department of Biology Washington University in St. Louis St. Louis Missouri; ^6^ Natural Resource Ecology Laboratory Colorado State University Fort Collins Colorado; ^7^ Department of Ecology, Evolution, and Marine Biology University of California Santa Barbara Santa Barbara California

**Keywords:** Imnavait Creek, minirhizotron, nitrogen, rhizon samplers, soil enzymes, Toolik Field Station

## Abstract

In addition to warming temperatures, Arctic ecosystems are responding to climate change with earlier snowmelt and soil thaw. Earlier snowmelt has been examined infrequently in field experiments, and we lack a comprehensive look at belowground responses of the soil biogeochemical system that includes plant roots, decomposers, and soil nutrients. We experimentally advanced the timing of snowmelt in factorial combination with an open‐top chamber warming treatment over a 3‐year period and evaluated the responses of decomposers and nutrient cycling processes. We tested two alternative hypotheses: (a) Early snowmelt and warming advance the timing of root growth and nutrient uptake, altering the timing of microbial and invertebrate activity and key nutrient cycling events; and (b) loss of insulating snow cover damages plants, leading to reductions in root growth and altered biological activity. During the 3 years of our study (2010–2012), we advanced snowmelt by 4, 15, and 10 days, respectively. Despite advancing aboveground plant phenology, particularly in the year with the warmest early‐season temperatures (2012), belowground effects were primarily seen only on the first sampling date of the season or restricted to particular years or soil type. Overall, consistent and substantial responses to early snowmelt were not observed, counter to both of our hypotheses. The data on soil physical conditions, as well interannual comparisons of our results, suggest that this limited response was because of the earlier date of snowmelt that did not coincide with substantially warmer air and soil temperatures as they might in response to a natural climate event. We conclude that the interaction of snowmelt timing with soil temperatures is important to how the ecosystem will respond, but that 1‐ to 2‐week changes in timing of snowmelt alone are not enough to drive season‐long changes in soil microbial and nutrient cycling processes.

## 1 INTRODUCTION

Climate warming is influencing Arctic tundra ecosystems by shifting the timing of seasonal events such as snowmelt and soil thaw (Ernakovich et al., 2014; MacDonald, 2010; Xu et al., 2013). This changing seasonality is leading to biological responses such as changes in plant phenology, timing of herbivore activity, and soil microbial and nutrient dynamics (Buckeridge, Banerjee, Siciliano, & Grogan, 2013; Høye, Post, Meltofte, Schmidt, & Forchhammer, 2007). Of these, aboveground responses are better studied than those belowground in soils, and furthermore, for soil responses, surprisingly little attention has been paid to the most likely future scenario: the combination of warming and earlier snowmelt (Callaghan et al., 2011; Chapman & Walsh, 2007; Wipf & Rixen, 2010). To understand the future of Arctic tundra ecosystems, we must understand how soil organisms (including plant roots, microbes, and soil fauna) and soil processes such as decomposition and nutrient cycling will respond to warming and an earlier onset of the growing season.

In Arctic tundra soils, decomposition controls both carbon stocks (Davidson & Janssens, 2006) and nutrient availability for plants and microbes (Schimel & Bennett, 2004). Decomposition in Arctic tundra soils is driven by saprotrophs (mainly bacteria and fungi) that enzymatically degrade plant litter and soil organic matter stocks, mineralizing nutrients that are then retaken up by plants and microbes (Burns & Dick, 2002; Schimel & Bennett, 2004). Higher trophic‐level organisms such as protists and microarthropods also contribute by shredding and digesting organic matter (Seastedt, 1984) or consuming saprotrophs, releasing nutrients (Clarholm, 1985). Plants take up some of these released nutrients and can also stimulate further decomposition by feeding C to microbes via rhizodeposition or transfer to mycorrhizae (Clemmensen, Michelsen, Jonasson, & Shaver, 2006; Kuzyakov, Friedel, & Stahr, 2000; Sinsabaugh & Moorhead, 1994; Weintraub, Scott‐Denton, Schmidt, & Monson, 2007). All of these soil organisms and processes exhibit pronounced seasonal timings that are likely to respond to changing seasonality.

**Figure 1:**
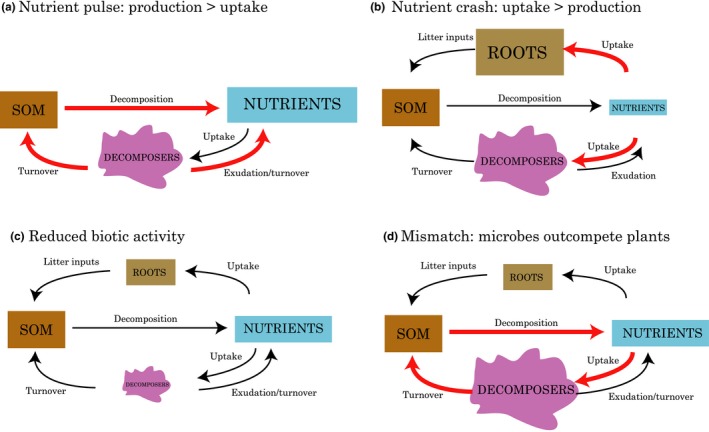
Four biogeochemical scenarios relevant to our hypotheses. Biogeochemical pools are shown as colored boxes with larger boxes indicating that that pool would be larger in that scenario than in others. Fluxes are shown as arrows, with elevated flux levels shown in red. (a) Over winter, nutrients build up in soils due to lack of plant uptake and substantial microbial activity below snow. This large pool is visible as a “pulse” of nutrients at the start of the season. (b) Midsummer, a “crash” in nutrients has been observed due to high plant and microbial demand for nutrients. (c, d) Two possible scenarios we might observe if plants are damaged by exposure to cold temperatures with early snowmelt. (c) If microbes are reliant on plant inputs (e.g., via rhizodeposition), all biotic activity may slow, leading to a delayed crash in nutrient availability. (d) If microbes continue to thrive, they may immobilize nutrients and take up C from damaged roots leading to a phenological “mismatch”

Nitrogen (N) is an essential plant macronutrient in Arctic tundra ecosystems, where it often, though not always, limits both plant production and microbial activity (Mack, Schuur, Bret‐Harte, Shaver, & Chapin, 2004; Melle, Wallenstein, Darrouzet‐Nardi, & Weintraub, 2015; Nowinski, Trumbore, Schuur, Mack, & Shaver, 2008; Weintraub & Schimel, 2003). The main source of N in Arctic soils is the large stocks of organic N, which can be mined enzymatically for amino acids and sugars, and other organic N monomers, which can ultimately be mineralized to NO_3_^−^ or NH_4_^+^ (McLaren, Darrouzet‐Nardi, Weintraub, & Gough, 2017). During the snow‐covered season (approximately September through May), N accumulates in soil bacteria and fungi, which then release it in a large pulse during soil thaw in association with microbial turnover (Figure 1a; Lipson, Schmidt, & Monson, 1999). Snow depth and the timing of melt can affect the size of this pulse by altering winter soil temperatures and patterns of N release in the spring (Buckeridge et al., 2013; Edwards & Jefferies, 2013). N cycling in Arctic soils is also dynamic during the summer growing season (McLaren et al., 2017), sometimes exhibiting a nutrient “crash” in which extractable NH_4_^+^, NO_3_^−^, and amino acids fall to undetectable levels midseason as plant and microbial usage of N peaks (Figure 1b; Weintraub & Schimel, 2005). In light of these strong seasonal patterns, it is important to understand how they will respond to changing seasonal timings such as earlier snowmelt and plant phenology.

Effects of changing seasonality in Arctic ecosystems have most commonly been investigated using *delayed* snowmelt experiments, typically accomplished by using snow fences that deepen snowpack and delay snowmelt, resulting in several weeks delay in plant phenology and increased water input (Borner, Kielland, & Walker, 2008; Rogers, Sullivan, & Welker, 2011; Wipf & Rixen, 2010). Soil N cycling also responds to snow fence treatments: Deeper snow warms the soil, which increases nutrient availability via stimulation of microbes (Buckeridge & Grogan, 2008; Freppaz, Williams, Seastedt, & Filippa, 2012; Schimel, Bilbrough, & Welker, 2004). Snow fences also trap wind‐blown litter, which could affect nutrient cycling as well (Fahnestock, Povirk, & Welker, 2000). Increased nutrient cycling has also been observed in an experiment where increasing snow depth was decoupled from delayed snowmelt (Natali, Schuur, & Rubin, 2012). A key question is whether we might see the reverse effect—that is, decreased microbial biomass and nutrient cycling activity with earlier snowmelt and soil exposure to colder air temperatures in the late spring.

In the case of *advanced* rather than *delayed *snowmelt, a multiyear snow removal experiment in a tussock tundra ecosystem near Toolik Lake, Alaska, showed that advanced snowmelt advanced the start of the growing season for plants as well as the onset of plant senescence (Khorsand Rosa et al., 2015; Starr, Oberbauer, & Pop, 2000). There was no strong evidence of a change in net C balance due to a shift—but not extension—of the growing season (Oberbauer, Starr, & Pop, 1998) and no clear effect on plant photosynthetic capacity (Starr & Oberbauer, 2003). Results from the same early snowmelt manipulation on which we focus in this study corroborate these findings, showing advanced aboveground plant phenology (Livensperger et al., 2016). However, plant responses to early snowmelt are not always the reverse of those with delayed snowmelt. In several cases from forested and alpine systems, early snowmelt treatments have been shown to damage plants due to the loss of the insulating snow layer (Wipf, Rixen, & Mulder, 2006; Wipf, Stoeckli, & Bebi, 2009). These negative effects have also been suggested by eddy flux tower data showing that years with earlier snowmelt do not necessarily have greater early‐season plant uptake of C (Humphreys & Lafleur, 2011). These effects in the plant community are linked to belowground effects in soil organisms, decomposition, and nutrient cycling, but no study to date has comprehensively connected these components in the context of advanced snowmelt.

**Figure 2:**
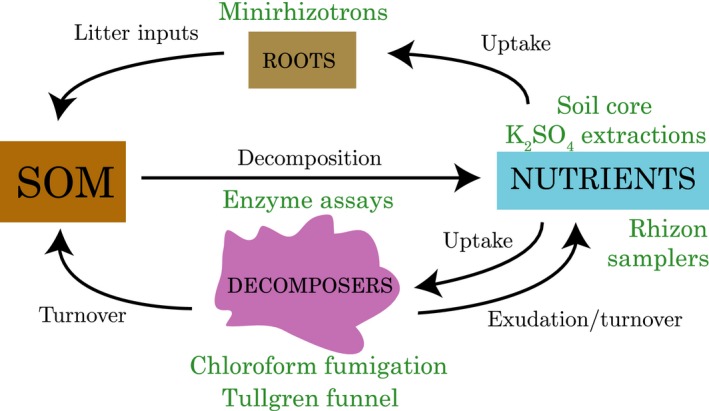
Belowground biogeochemical pools (colored boxes), fluxes (black arrows), and measurement techniques used in this study (green)

Based on the contrasting evidence for advancement of plant phenology versus damage of plants via loss of snow insulation in early snowmelt experiments, we generated two alternative hypotheses about how the belowground portions of the ecosystem would respond to early snowmelt: (a) early snowmelt, particularly coupled with warming, advances the timing of root growth, microbial activity, and N cycling processes, including the nutrient pulse at snowmelt and the “crash” during peak plant physiology; (b) loss of insulating snow cover damages plants, leading to reduced root growth, with various possible outcomes. These outcomes could include reduced stimulation of microbes via rhizodeposition leading to overall reduced biotic activity and a delay in the nutrient crash (Figure 1c); or even a phenological “mismatch” in which microbes take up carbon from damaged roots and outcompete plants for N leading to greater N immobilization in microbes (Steltzer, Landry, Painter, Anderson, & Ayres, 2009; Figure 1d). Loss of insulation could also affect the microbes directly; little is known about how these factors might interact. To test these hypotheses, we set up an early snowmelt × warming field manipulation experiment in an Arctic tundra ecosystem in which we monitored physical conditions, plant phenology, root growth, microbial biomass, soil enzyme activities, microarthropod densities, and soil nutrient dynamics (Figure 2). With these data, we assess the effects of early snowmelt on three main components of the soil biogeochemical system: plants, decomposers, and soil nutrients.

## 2 MATERIALS AND METHODS

### 2.1 Study site

**Figure A1:**
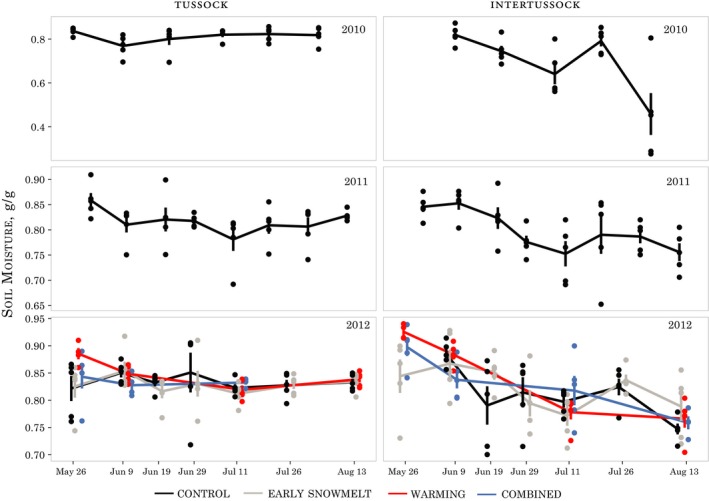
Gravimetric soil moisture from soil cores over three years of the experiment. Mean ± SE by treatment (n = 5) are shown as bars.

Our study site (68°37'37" N, 149°19'11" W) is 2.3 km south–southwest of the intersection of Imnavait Creek and the Dalton Highway, east of Toolik Field Station, on the north slope of the Brooks Range in Alaska, USA. The plant community is moist acidic tundra (MAT), a common vegetation type in the northern foothills of the Brooks Range dominated by *Eriophorum vaginatum*, a tussock forming sedge (Shaver & Chapin, 1991). Surrounding the tussocks are a mix of mosses, other sedges such as *Carex bigelowii*, evergreen shrubs such as *Vaccinium vitis‐idaea*, and deciduous shrubs such as *Betula nana* and *Salix pulchra*. There is an uneven cover of organic soil 0–20 cm thick in MAT (Shaver & Chapin, 1991). The organic layer directly underneath *E. vaginatum* plants consists primarily of decaying roots of *E. vaginatum*. The soil organic matter has a mean C concentration of 45%, N concentration of 0.8%, and pH of 4.4. Due to low evapotranspiration and melting ice in the active layer of these permafrost soils, soil moisture in this ecosystem remains relatively high and constant at a level of ~0.8 g water/g wet soil (Figure A1; Weintraub & Schimel, 2005).

### 2.2 Early snowmelt and warming treatments

**Figure 3:**
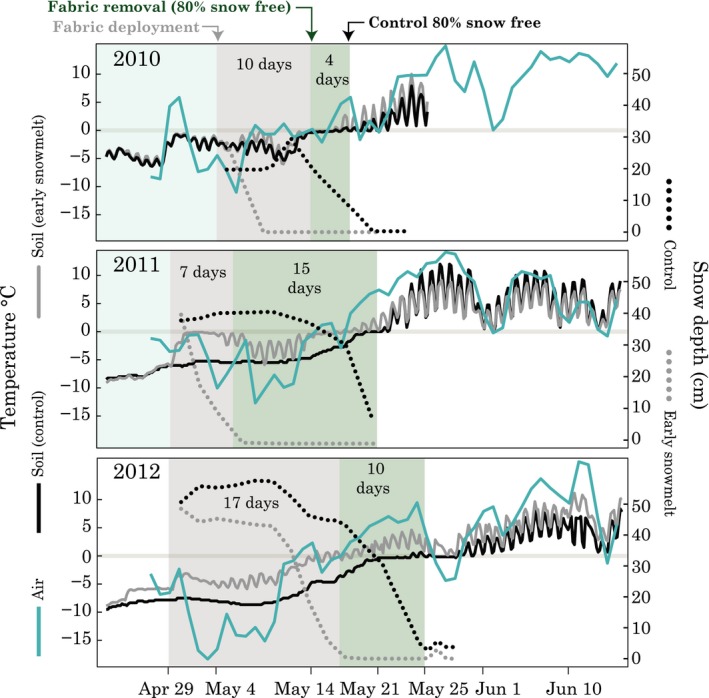
Timing of fabric deployment, snowmelt, snow depth, air temperature, and soil temperature in control and early snowmelt plots. Air temperatures are daily means; soil temperatures were recorded every 4 hrs with iButtons buried at 10 cm depth in *E. vaginatum* tussocks. iButtons were removed earlier in 2010; hence, there are lack of data in late May and June

**Figure A2:**
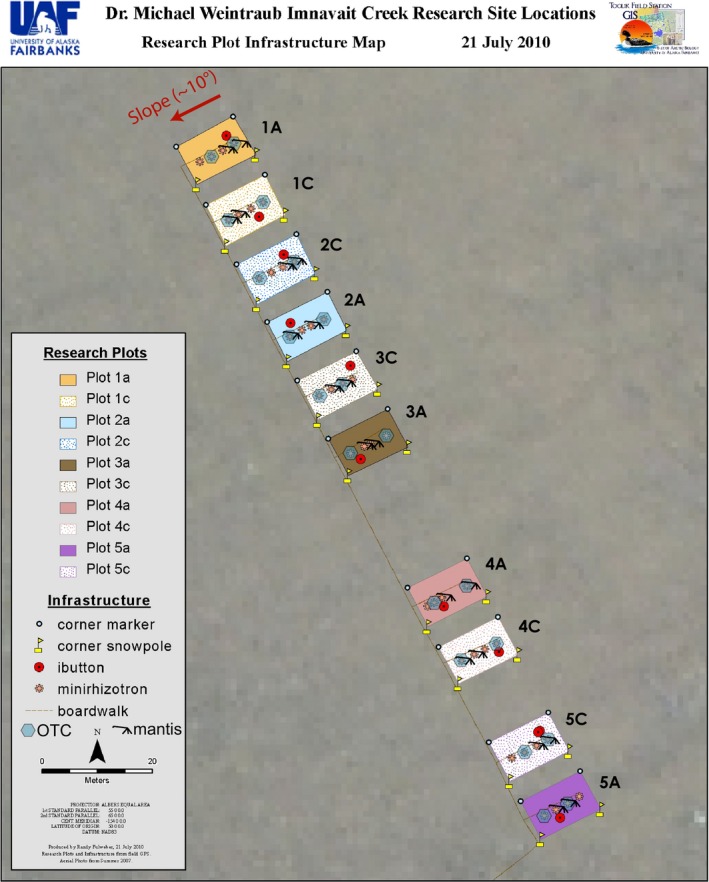
Plot map created by the Toolik Field Station GIS team, modified to show OTC and mantis locations on our experimental plots (68° 37' 37" N, 149° 19' 11" W). Mantises are our instrument arrays containing soil and air temperature, soil moisture, and NDVI. The plots are near the ridge top, but on a mild slope (~10°) with long side of the plots oriented parallel to the slope, the boardwalk being the downhill side.

**Figure A3:**
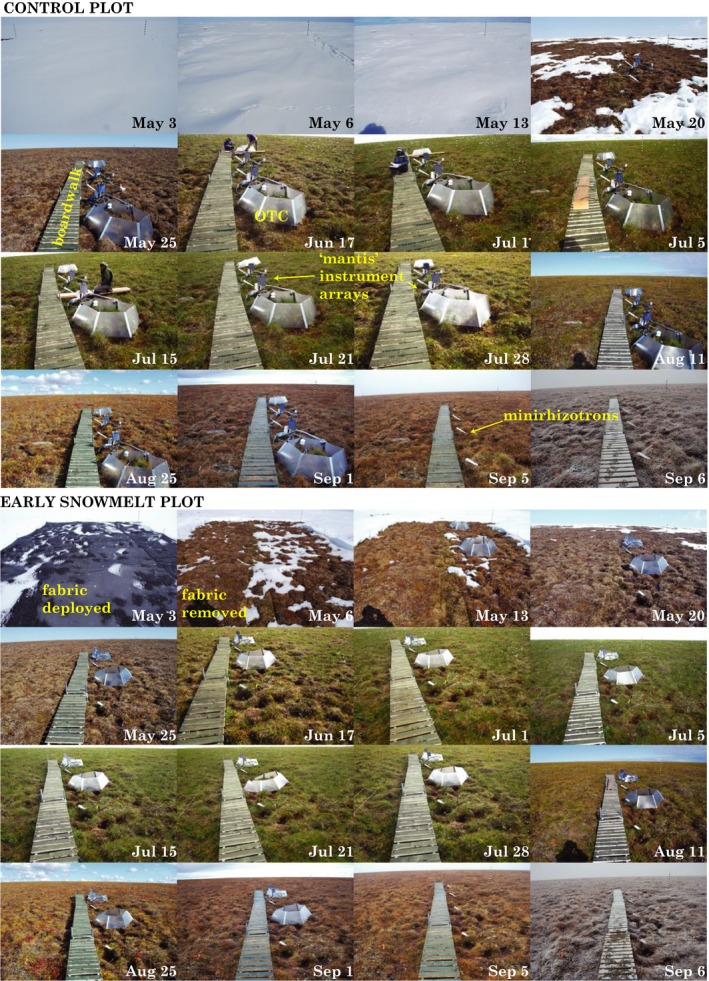
Plot photos—control on top, early snowmelt on bottom—over the course of the 2011 season. Times series of one plot from each treatment are shown, with photo date in white. The 50% shadecloth fabric is visible in the first early snowmelt photo. Other plot features that are visible include boardwalk to reduce plot trampling damage, the ITEX‐style OTCs, instrument arrays for monitoring air and soil conditions, and the small white minirhizotron tubes. For plot scale, see Figure S1 as well as research team members visible in several photos. All photos were taken from the site‐wide stretch of boardwalk on the downhill side of the plots. The boardwalk spurs that are visible in these photos extend into the plots from the site‐wide stretch.

**Figure A4:**
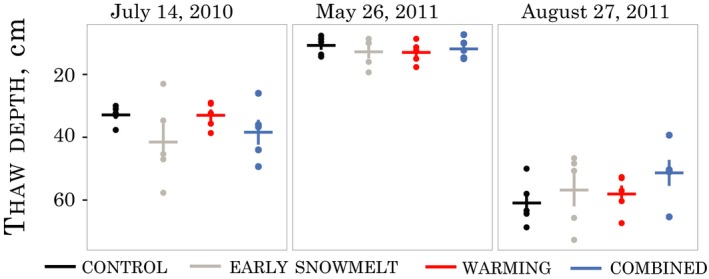
Thaw depth on three dates over the first two years of the experiment. Mean ± SE by treatment (n = 5) are shown as bars, with each point indicating the average of 3 measurements with a metal probe.

We used a snowmelt advancement technique that isolates changes in snowmelt timing by using shade cloth (Steltzer et al., 2009) and crossed it with a standard open‐top chamber (OTC) warming treatment (Oberbauer et al., 2007). In August 2009, we laid out five blocks of two 8 × 12 m plots at our study site (Figure A2). The plots were demarcated in the season prior to applying the treatments in order to install minirhizotron tubes for root growth measurements and bury iButton temperature loggers (Maxim, San Jose, California, USA) in the soil prior to snowfall. The plots were parallel in arrangement, with 5–30 m between blocks and 4 m between paired plots (Figure A2). At the beginning of each of our study years (2010–2012), we applied early snowmelt and warming treatments. One of the plots in each block was randomly chosen for the early snowmelt treatment. Early snowmelt was achieved by deploying 8 × 12 m pieces of black 50% shade cloth fabric (Steltzer et al., 2009) until plots were snow‐free (Figure A3; we defined snow‐free as <20% snow cover). This snowmelt manipulation technique accelerates snowmelt but does not alter the amount of melting snow or increase thaw depth (Livensperger et al., 2016; Steltzer et al., 2009). The fabric deployment date was chosen to achieve a target snowmelt advancement of 14 days. In 2010, we deployed the fabric on May 4, resulting in an advancement of 4 days. To achieve a longer advancement in 2011, we deployed the fabric on April 29, resulting in an advancement of 15 days. We deployed on the same day of year in 2012 (April 28 due to leap year), resulting in an advancement of 10 days. The control plots became snow‐free on 18, 21, and 26 May in the 3 years, respectively (Figure 3). Snow depth was regularly monitored during snowmelt using a metal rod. Thaw depth was also monitored later in the season with the same implement, though only on three occasions (Figure A4).

In each of the 10 plots, four 1 × 1 m subplots were defined by the locations of the minirhizotron tubes, which were arrayed at equally spaced intervals from the top and bottom edges (8 m sides) of the plots. In each plot, two of the subplots were randomly assigned to the warming treatment. Warming was achieved by deploying International Tundra Experiment‐style passive warming OTCs (Marion, 1989, Sullivan & Welker, 2005). Made from Sun‐Lite HP fiberglass (Solar Components, Manchester, NH, USA), our chambers were hexagonal in shape, 1.5 m in basal diameter, 40 cm in height, and had a 60° wall angle. OTCs were deployed as soon as the subplots were sufficiently snow‐free to allow placement of the chamber, which typically was the same date that plots first met the 80% snow‐free criterion. OTCs were left in place until 2 September 2010, 5 September 2011, and 15 August 2012. Soil temperatures were measured using iButtons buried in each plot at 10 cm depth in *E. vaginatum* tussocks and in intertussock spaces.

### 2.3 Aboveground plant phenology—broadband NDVI trajectories

**Figure A5:**
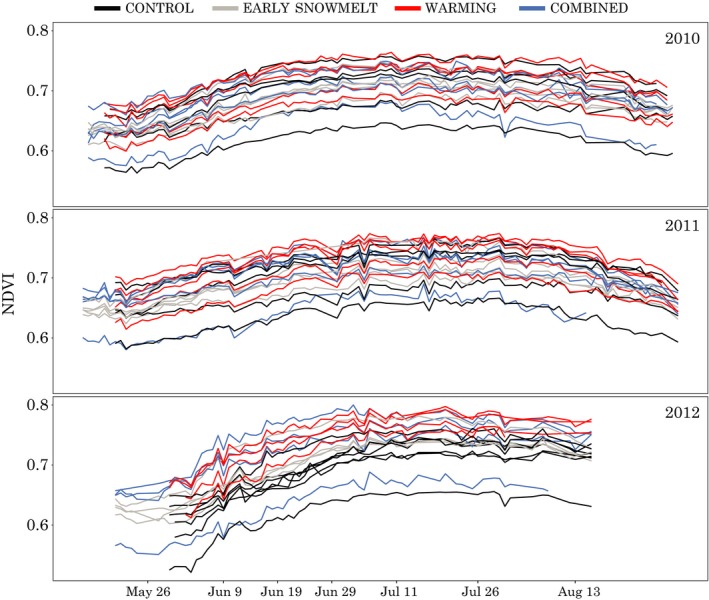
Raw NDVI data. Each line indicates the time series of NDVI readings from each of our 20 NDVI sensors. Vertical displacement of the seasonal trends is largely due to differences in the ground cover underneath the sensors (i.e., relative abundance of rock vs. plant vs. soil).

Leaf area development was monitored using near‐surface broadband NDVI, which was measured using two radiation sensors mounted at ~50 cm height, recording a circular area of ~0.75 m^2^ (Sweet, Griffin, Steltzer, Gough, & Boelman, 2015). The sensors recorded (a) photosynthetically active radiation (PAR, 400–700 nm; PAR Smart Sensor; Onset Computer Corporation) and (b) the shortwave (infrared and visible) spectrum (300–1,100 nm). Data were collected during the 2 hrs surrounding solar noon (1,200–1,400 AKST) during cloud‐free periods. Broadband NDVI was calculated following Huemmrich, Black, Jarvis, McCaughey, and Hall (1999), with further rationale and calculation details available in Sweet et al. (2015). To compare season‐long trends in broadband NDVI among treatments, we scaled the time series on a per‐sensor basis (20 in total) to have a standard deviation of 1 and centered each series by subtracting the average of their first four readings, which represent an early‐season baseline during which all plots exhibited their post‐snowmelt minimum. We term the output of this scaling process “broadband NDVI trajectories” because they emphasize seasonal trends while removing the effects of plot‐to‐plot variation in overall broadband NDVI magnitude, which is driven mainly by how much nonvegetative material is in the viewing area (rock, soil, etc.). The unmodified broadband NDVI time series are included in the Supporting Informations for reference (Figure A5). The offset in magnitudes among sensors visible in these unmodified series demonstrates why we took this per‐sensor scaling approach.

### 2.4 Aboveground plant phenology—marked individuals

To put our belowground results in context, we compare the timing of root growth to the timing of first leaf expansion on individual plants in each of the treatment plots. Data from the 2012 season were included in Livensperger et al. (2016). We use the timing of first leaf expansion because it was our most consistently recorded variable at the beginning of the season, and closest to the timing of the snowmelt manipulation. Not all plants had an obvious flowering time in all years, but leaf expansion was consistently noted among the species we observed and so provides the best indication of overall early‐season plant phenology at our study site. We focused on four of the dominant species: the tussock forming sedge *E. vaginatum*, the sedge *C. bigelowii*, and two woody species, *B. nana* and *S. pulchra*. In one warmed OTC subplot and one unwarmed subplot in each of our 10 plots (five early snowmelt and five control), we marked five representative individuals of each species using small colored wire and plastic beads. Phenological observations were made every 2–3 days during May and June of each of our three growing seasons. First leaf expansion was defined as the time at which newly grown leaves from each marked individual were fully unfurled and had reached full size. *Eriophorum vaginatum *is evergreen and so the first new leaf of the year to reach 4 cm in length was recorded, while the other three plants are deciduous making first leaf expansion an easily observable event.

### 2.5 Root production

**Figure A6:**
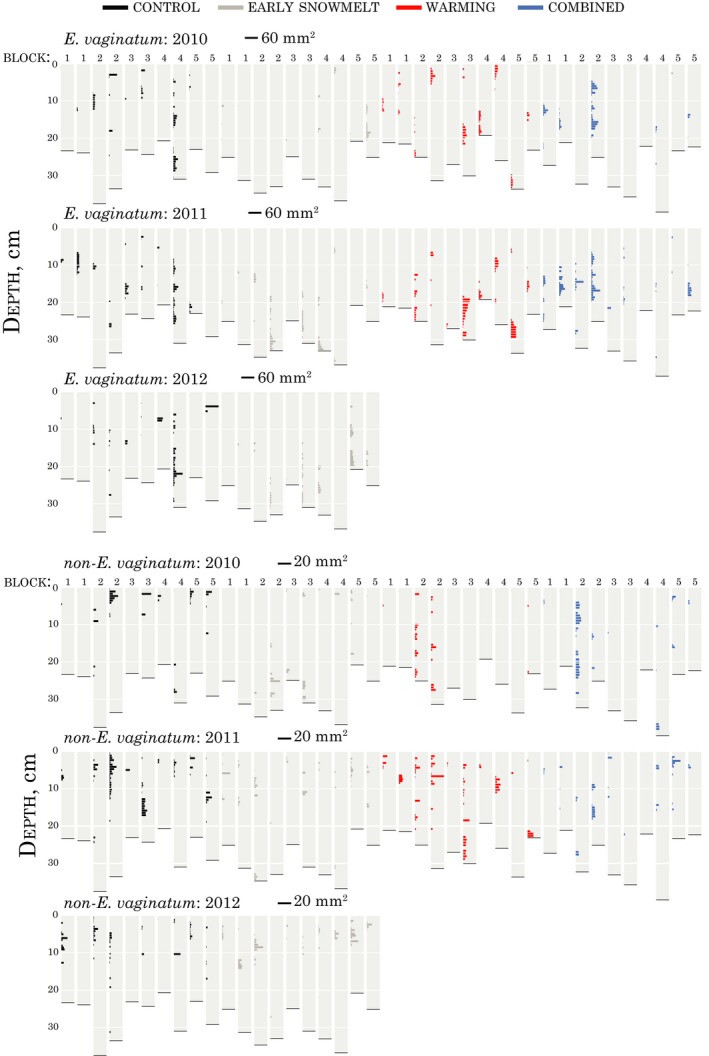
Root biomass at end of each season (August 11, 2010, August 13, 2011, August 4, 2012). Upper panels are E. vaginatum and lower panels are non‐E. vaginatum. Each vertical gray subplot indicates a minirhizotron tube, the observed root area shown on the horizontal axis at each depth. The black lines at the bottom of each tube indicate the maximum observation depth of each tube. Note the different scales between upper and lower panels due to higher maximum root biomass in E. vaginatum.

Root production was estimated with a BTC‐2 minirhizotron camera system (Bartz Technology, Carpinteria, CA, USA). In August 2009, we installed minirhizotrons in four subplots (two warmed by OTC and two unwarmed) in each of our 10 plots (40 total). Minirhizotrons were installed approximately 45° to the ground surface to an average vertical depth of 26 cm. The exact angle of each tube was measured to 0.1 of a degree, and the depth of each tube was adjusted accordingly. A grid of 0.9 cm × 1.3 cm rectangles was etched on the wall of the minirhizotrons to easily identify sampling locations from week to week (average: 42 locations/minirhizotron). This grid was oriented facing directly up. The portion of tube protruding from the ground was painted white to reflect solar radiation and prevent warming of the adjacent soils. Removable foam insulation was placed in each tube when not being sampled. The lower end was permanently sealed using a rubber stopper, while a removable cap was placed on the upper end. Images were collected 29 times at approximately 1‐week intervals over the 2010–2012 growing seasons. Due to extensive soil core collections within the small footprint of the OTCs in 2012, only the control and early snowmelt tubes were monitored in 2012. Roots showed indications of delayed colonization of the minirhizotrons (Iversen et al., 2012) in 2010, casting some doubt upon the measured values during that year; thus, we excluded these from analysis. Root images were analyzed using Rootfly (Version 1.8.36; Clemson University, 2005–2010). The length and width of new root growth were digitized and encoded as a data table. Images captured a larger soil area than the grid, but only roots present within the grid lines were analyzed. To estimate root production in units of g/m^2^, we assumed a 3 mm depth of field (Sullivan, Arens, Chimner, & Welker, 2008; Sullivan et al., 2007; Sullivan & Welker, 2005). Fine root projected area was then converted to root mass using regressions developed separately for *E. vaginatum* and for other species (Sullivan et al., 2007). Estimates of root mass were then scaled to one square meter. Though the tube depths varied (Figure A6), this is indicative of the natural variation in depth to permafrost. All tubes were installed to maximum thaw depth during their August installation. Tubes stayed in their original positions throughout the experiment, with no appreciable heave effects observed. Even if present, minor heave effects would be more likely to occur outside of the growing season and thus unlikely to affect our growing season production measurements. We found some evidence of under‐sampling of *E. vaginatum* roots in the top 10 cm; in many tubes, root areas in the top 10 cm were lower than the 10–20 cm values. This may have been due to the positioning of the minirhizotrons with respect to the *E. vaginatum *tussocks. To account for this, we repeated our root analyses using only the 10–20 cm depth. Trends and results were similar and so here we present only the full profile data. The vertical trends within tubes are shown in Figure A6.

### 2.6 Soil pore water nutrients

We measured soil pore water nutrient concentrations nondestructively at high temporal resolution using microlysimetry allowing us to gain insight into nutrient processes that are typically not observable due to concerns over destructive sampling. High temporal resolution is important because studies that are restricted to one or several measurements per year may miss important seasonal events (Darrouzet‐Nardi & Weintraub, 2014; Weintraub & Schimel, 2005). Microlysimeters (rhizon samplers, Eijkelkamp Soil and Water, part no. 192101) were used to make nondestructive measurements of soil pore water labile N (NH_4_^+^, NO_3_^−^, and amino acids quantified as total free primary amines [TFPA]). We installed one microlysimeter in a tussock microsite (within an *E. vaginatum *tussock) and one in an intertussock microsite in each of our four treatments (control, early snowmelt, warming, and combined) within our five experimental blocks, for a total of 40 microlysimeters. The microlysimeters are 10‐cm‐long, 2.5‐mm‐diameter porous PVC tubes that are capped on the bottom and connected to a plastic tube on the top with a needle that is left above ground. The microlysimeters were sampled by attaching an evacuated 6‐ml tube sealed with a rubber septum (Greiner Vacuette No. 456089) to the needle and leaving them for 1–12 hr, depending on how rapidly soil water filled the tube. After collection, microlysimeter samples were frozen for later nutrient analysis (described below). Microlysimeters were sampled every 3–5 days from 2 June–13 August 2010; 21 May–17 September 2011; and 17 May–17 August 2012. In total, there were 25 measurement dates in 2010, 34 in 2011, and 28 in 2012. Around 10% of the tubes did not yield enough soil pore water for analysis, leaving the final total of samples analyzed over three seasons at 3,105.

### 2.7 Soil cores

While the nondestructive measurements for plants and soil pore water were taken for 3 years, measurements requiring destructive soil sampling were made in the final year of the study to avoid damaging the plots. We collected soil core samples for nutrient, microbial biomass, and plant tissue analysis every 10–14 days during the summer of 2012. We sampled tussock (within an *E. vaginatum *canopy) and intertussock locations within the plots in the four treatments (control, early snowmelt, warming, and combined). Collection began with spring thaw and continued until plant senescence, for a total of seven sampling dates in 2012 (25 May–14 August). Due to limited space in the warming treatment's OTCs, those treatments were sampled on only four of the seven dates. In total for 2012, 295 samples were collected. Each sample was cored with a 5‐cm‐diameter metal soil corer to a depth of at least 10 cm. Within 2 hrs of collection, live plant material was removed and the top 10 cm of organic soil was homogenized by hand. Once homogenized, a 5 g (wet weight) subsample of each soil was shaken with 25 ml 0.5 M K_2_SO_4_ in 50‐ml centrifuge tubes on an orbital shaker table at ~120 rpm for 1 hr, then vacuum‐filtered through Millipore APM 15 glass fiber filters. The extracts were then frozen for nutrient analysis. A second 5 g subsample was put into a 250‐ml Erlenmeyer flask for measurement of microbial biomass. A third 5 g subsample was used to measure water content of the soil‐by‐mass difference between fresh and oven‐dried (60°C) soil. Samples were also collected in control conditions just outside the plots during the 2010 and 2011 seasons. Though not presented here, those data are used to inform one analysis of early‐season differences (Table 1).

**Table 1:** Snow‐season soil temperatures (separated into late summer, fall, and winter; 10 cm depth, measured with iButtons) and soil N cycling measurements during the subsequent spring thaw periods

Year	Late summer soil temperature °C 18 August–17 September	Fall soil temperature °C 18 September–31 December	Winter soil temperature °C 1 January–30 April	Snow depth before melt (cm)
2009–2010	3.2	−3.4	−10.7	30 ± 2
2010–2011	5.4	−2.8	−7.8	40 ± 2
2011–2012	3.2	−3.8	−11.7	58 ± 2

Year	Tussock Labile N at thaw µg N/g soil	Tussock MBN at thaw µg N/g soil	Tussock LAP activity at thaw nmol g^−1^ soil hr^−1^	Tussock NAG activity at thaw nmol g^−1^ soil hr^−1^
2010	13.8 ± 1.2	140 ± 14	0.6 ± 0.4	234 ± 30
2011	32.4 ± 7.4	300 ± 60	39 ± 2.5	468 ± 74
2012	29.0 ± 11.8	130 ± 30	7.0 ± 1.5	437 ± 94

Year	Intertussock Labile N at thaw µg N/g soil	Intertussock MBN at thaw µg N/g soil	Intertussock LAP activity at thaw nmol g^−1^ soil hr^−1^	Intertussock NAG activity at thaw nmol g^−1^ soil hr^−1^
2010	17.4 ± 3.2	244 ± 46	12.4 ± 2.0	133 ± 13
2011	45.7 ± 12.5	318 ± 37	38.2 ± 2.3	162 ± 23
2012	13.2 ± 4.7	234 ± 18	12.5 ± 5.1	151 ± 27

Note. Soil cores were taken shortly after soil thaw (snowmelt dates were 18, 21, and 26 May in the 3 years, respectively). The transition from late summer to fall (18 September) was defined by soil freezing. Values shown are mean ± *SE*. In 2010–2011, warmer fall and winter temperatures were associated with higher labile N, microbial biomass N (MBN), leucine aminopeptidase (LAP) activity, and *N*‐acetyl glucosaminidase (NAG) activity in the following growing season. 2012 numbers are shown in Figures 10 and 11. 2010 and 2011 data are from cores taken just outside of the control plots and representative of control conditions.

### 2.8 Microbial biomass

Microbial biomass carbon and nitrogen (MBC and MBN) in the soil core samples were quantified using a direct‐chloroform‐addition modification of the chloroform fumigation‐extraction technique (Brookes, Landman, Pruden, & Jenkinson, 1985; Scott‐Denton, Sparks, & Monson, 2003). In brief, 5 g (wet weight) of soil was combined with 2 ml of ethanol‐free chloroform and incubated at room temperature for 24 hr in a stoppered 250‐ml Erlenmeyer flask. Following incubation, flasks were vented in a fume hood for 30 min and extracted and frozen as described above. Fumigated extracts were analyzed for total organic carbon (TOC) and total dissolved N (TDN) concentrations on a Shimadzu analyzer (TOC‐V_CPN_; Shimadzu Scientific Instruments Inc., Columbia, MD, USA). MBC and MBN were calculated as the difference between TOC and TDN concentrations extracted from fumigated versus nonfumigated samples. No extraction efficiency correction factor (*k*_EN_) was applied, as it is unknown for these soils, so values presented represent only extractable microbial biomass C and N. The nonfumigated extractions were the same K_2_SO_4_ extractions that were used for nutrient analyses.

### 2.9 Soil microarthropods

We estimated densities of soil‐dwelling microarthropods on five dates in 2012 coinciding with five of the soil core collection dates (7 June–26 July). Microarthropods were collected only in control and early snowmelt plots due to concerns about plot disturbance in OTCs. Microarthropods were heat‐extracted from soil samples into a solution of 90% ethanol using Berlese–Tullgren funnels (Moore, Tripp, Simpson, & Coleman, 2000) over a period of 5 days. Numbers of Collembola, Oribatid mites (both of which are detritivorous and fungal feeding), and predatory mites were counted from each sample, and then, we divided the number of individuals in each group and the sum of all three groups from each soil sample by the volume of the soil sample (cm^3^) to calculate the final reported densities.

### 2.10 Soil enzymes

Soil N‐acquiring hydrolytic enzyme activity assays were conducted using high‐throughput microplate assays (Melle et al., 2015; Rinkes, Weintraub, DeForest, & Moorhead, 2011; Saiya‐Cork, Sinsabaugh, & Zak, 2002; Wallenstein, McMahon, & Schimel, 2009). Fluorogenic substrates at saturating concentrations (200 µM) were used to measure activities of two hydrolytic N‐acquiring enzymes: *N*‐acetyl‐β‐glucosaminidase (NAG) and leucine peptidase (LAP), and one hydrolytic C‐acquiring enzyme, β‐glucosidase (BG). Activity rates were calculated by comparison with 100 µM standards of the fluorogenic reaction product (4‐methyl umbelliferone for NAG and BG; 7‐amino‐4‐methylcoumarin for LAP) after correcting for quenching by the soil matrix (fluorogenic product + sample). A colorimetric assay using 2 mM ABTS (also saturating) was performed for oxidative enzyme activity (specifically, phenoloxidases). Phenoloxidase activities were calculated using a micromolar extinction coefficient of 21.9/µmol. Enzyme assays were conducted in 96‐well (2 ml well size) microplates, with four replicate wells per sample. Sample slurries were prepared by blending 2.7 g of wet soil with 91 ml of 50 mM sodium acetate buffer for 2 min using a conventional kitchen blender. Buffer pH was set to 5.0 as an approximation of field pH in MAT soils. Soil slurries were constantly mixed, while 800 μl aliquots of slurry were pipetted into the deep‐well plates using large orifice pipette tips. Two hundred microliters of the fluorogenic substrates was then added to these wells. Microplates were incubated at 10°C in the dark for 4 hr following substrate addition. Following incubation, 200 µl aliquots of the samples were pipetted out of each well and transferred to opaque black 96‐well microplates (hydrolytic enzymes) or clear microplates (phenoloxidase). Plates were read using a Bio‐Tek Synergy HT microplate reader (Bio‐Tek Inc., Winooski, VT, USA) with 365‐nm excitation and 460‐nm emission filters (hydrolytic). The ABTS‐based phenoloxidase assays were read at a wavelength of 420 nm. Enzyme activities were expressed as nmol reaction product hr^−1^ g^−1^ dry soil. For further details on the enzymes and the procedure, see Melle et al. (2015).

### 2.11 Nutrient analyses

Soil nutrient analyses for soil pore water and soil core extracts were conducted following Rinkes et al (2011). NH_4_^+^, NO_3_^−^, and TFPA (likely to be mostly amino acids) were analyzed using colorimetric (NO_3_^−^ and NH_4_^+^) or fluorometric (TFPA) microplate assays. NH_4_^+^ concentrations were measured using a modified Berthelot reaction (Rhine, Sims, Mulvaney, & Pratt, 1998). NO_3_^‐^ was measured using a modification of the Griess reaction (Doane & Horwath, 2003), which involves the reduction of nitrate to nitrite followed by colorimetric determination of nitrite. TFPA was measured using *O*‐phthaldialdehyde and mercaptoethanol (Darrouzet‐Nardi, Ladd, & Weintraub, 2013; Jones, 2002). Absorbance and fluorescence values were determined on a Bio‐Tek Synergy HT microplate reader (Bio‐Tek Inc.). Soil pore water collected with the microlysimeters was analyzed for NH_4_^+^, NO_3_^−^, and TFPA. All pore water concentrations are reported as µmol/L soil water (µM). An in‐depth discussion of the differences among the collection techniques (soil core extracts vs. microlysimeters) in the tussock control plots in 2011 can be found in Darrouzet‐Nardi and Weintraub (2014).

### 2.12 Data analysis

The effect of the early snowmelt, warming, and combined treatments was evaluated by evaluating the effect size of these treatments on each variable of interest (Cumming, 2013; Nakagawa & Cuthill, 2007). For most measured variables, effect sizes are reported as treatment/control ratios (denoted *T*_r_) with 95% confidence intervals estimated by bootstrapping (Carpenter & Bithell, 2000). In cases where ratios are not appropriate, such as the day of year for phenological events, differences between the treatments (treatment–control) are calculated, also with 95% bootstrapped confidence intervals. We calculated effect sizes for each measurement date, allowing us to evaluate changing effects of the treatments throughout the season using focused comparisons with the control treatment as opposed to pooling our nonhomogenous variances across the whole season (Rosenthal & Rosnow, 1985). For several of the more frequent measurements (soil pore water nutrients and NDVI), we also used seasonal averages based on the mean values across multiple dates, retaining the replication level of *n* = 5 for each treatment. Among all analyses, the only exception to the *n* = 5 level of replication is the minirhizotron root biomass data in which two minirhizotron tubes per plot were treated as independent for a total of *n = *10. We made this decision because the spatial distances between tubes in the same plots were similar to those between plots (Figure A2), no substantial blocking effects were observed, and high variation among tubes in the same plots (Figure A6) made plot averages undesirable.

## 3 RESULTS

### 3.1 Physical conditions

**Figure A7:**
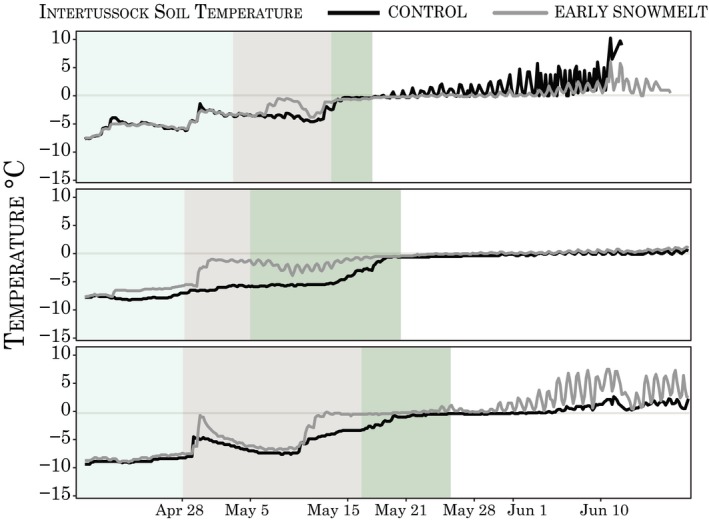
Complement to Figure 4 showing the intertussock soil temperatures recorded by iButton. The averages of 5 iButtons in each treatment are shown. Shading of dates matches that in Figure 4 for comparison purposes.

The fabric treatment advanced snowmelt by 4, 15, and 10 days in 2010, 2011, and 2012, respectively (Figure 3). In 2010, air temperatures near 0°C following fabric deployment followed by warm temperatures just after fabric removal accompanied the relatively small advancement period of 4 days. In 2011, warm air temperatures during fabric deployment melted the snow on the early snowmelt plots rapidly, but then, low temperatures following fabric removal (with daily means as low as −12°C) led to our longest snowmelt advancement: 15 days. In 2012, subzero temperatures during fabric deployment prevented melt and led to a longer fabric deployment. 2012 also had the deepest snowpack of the 3 years (50 ± 1 cm, vs. 24 ± 2 cm in 2010 and 39 ± 1 cm in 2011). After this cold period, warmer temperatures melted both control and treatment plots, ultimately leading to an intermediate advancement period of 10 days. In all 3 years, snowmelt date and soil thaw date were closely linked in the control plots, with above‐zero temperatures occurring in tussocks within several days of snowmelt each year (Figure 3). Intertussock soils thawed shortly thereafter (Figure A7). The fabric treatment warmed soils while it was deployed; it also caused daily oscillations in belowground temperature, which were clearly visible in 2011 and 2012 (Figure 3). The length of the transition from ice to water during which soils were at 0°C (isothermal period) varied among years, with a rapid thaw period in 2011 and a longer thaw period in 2012. Finally, the OTCs warmed the air by an average of 1.6°C in 2010, 1.9°C in 2011, and 1.4°C in 2012. Though sporadic, our three thaw depth measurements did not reveal clear differences in thaw depth among treatments (Figure A4).

### 3.2 First leaf expansion in marked individuals

**Figure 4:**
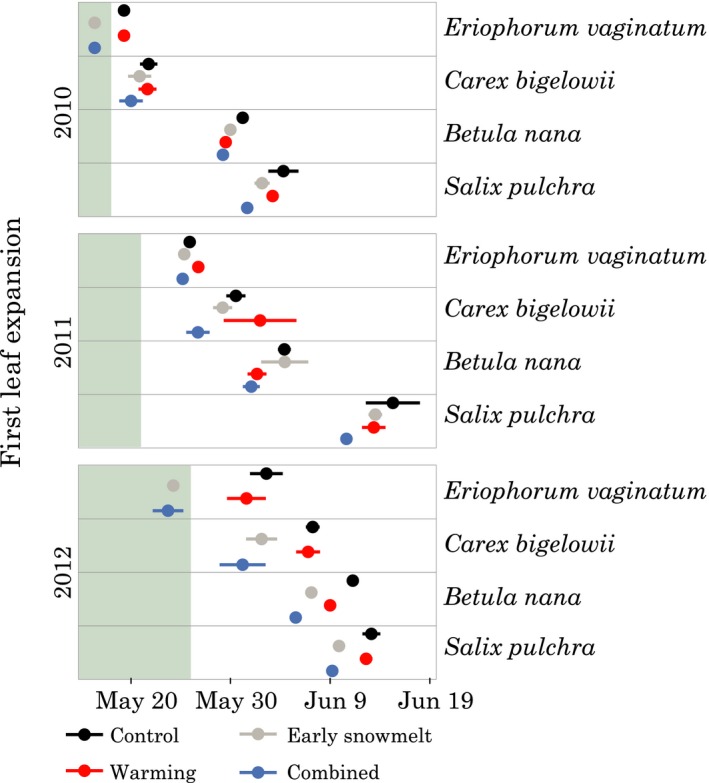
Mean date of first leaf expansion ± *SE* by treatment (*n* = 5). The transition from >20% snow cover in the controls to snow‐free is shown by the shading in the same way as Figure 3

In 2010, the short snowmelt advancement of 4 days slightly advanced first leaf expansion (Figure 4). The warming and early snowmelt treatments had similar effects on *B. nana*, but not on the other three species. In 2011, despite the longer snowmelt advancement of 15 days, a comparably large advance in first leaf expansion was not observed: *E. vaginatum* showed little difference among treatments (treatment–control [95% bootstrap CI] = −1 [−1, 0] days); *C. bigelowii* leafed out slightly earlier than the control in the early snowmelt treatment (−1 [−3, 0] days), and by a few days in the combined treatment (−4 [−6, −2] days); the two shrub species *B. nana* and *S. pulchra* showed similar treatment effects to *C. bigelowii*. The small treatment effect relative to the length of snowmelt advance in 2011 (15 days) coincided with the cold temperatures observed during the snowmelt advancement period. In 2012, although the snowmelt advance was shorter than in 2011 (10 days), a stronger treatment effect was observed. Effects in both early snowmelt and combined treatments ranged from advances in first leaf expansion of −3 [−6, −2] days for *S. pulchra* in the early snowmelt treatment to −10 [−12, −8] days for *E. vaginatum* in the combined treatment. The warming treatment alone showed a smaller effect for *B. nana* (−2 [−3, −2] days) and *E. vaginatum* (−2 [−4, 0] days), and no evidence of an effect in *C. bigelowii* or *S. pulchra*.

### 3.3 NDVI‐based assessment of phenology

**Figure 5:**
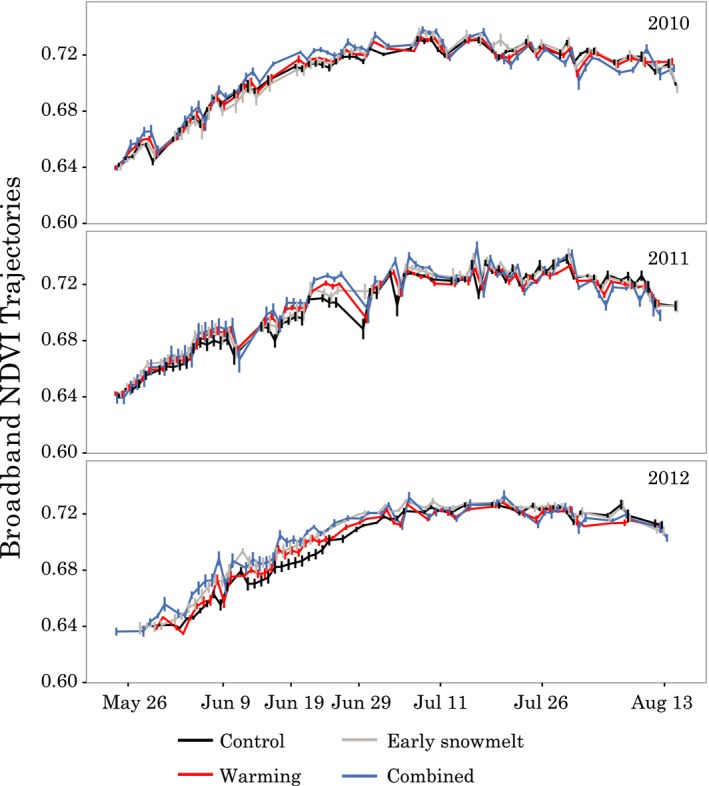
Scaled broadband NDVI trajectories from 20 “mantis” instruments employing radiation sensors with a ~0.75 m^2^ viewing area (mean ± *SE*). Treatments were measured simultaneously but are offset slightly to avoid overplotting

In 2010 and 2011, we saw relatively small differences in broadband NDVI trajectories among treatments, while in 2012, differences were larger (Figure 5). In examining broadband NDVI averages across the month of June (representing the period leading up to peak physiology), the largest differences in all years were in the combined treatment (combined–control [95% CI], June 2010: 0.007 [0.003, 0.011], June 2011: 0.010 [0.000, 0.020], June 2012: 0.016 [0.009, 0.022]). In 2012, all three treatments showed subtle but detectable increases in NDVI relative to the controls in the average June values (early snowmelt: 0.011 [0.005, 0.018], warmed: 0.007 [0.001, 0.012], combined: 0.016 [0.009, 0.022]). During and after peak NDVI (evaluated as July averages), treatment differences dropped below detection (e.g., July averages: early snowmelt: 0.003 [0, 0.006], warmed: −0.001 [−0.003, 0.001], combined: 0 [−0.004, 0.005]).

### 3.4 Root production

**Figure 6:**
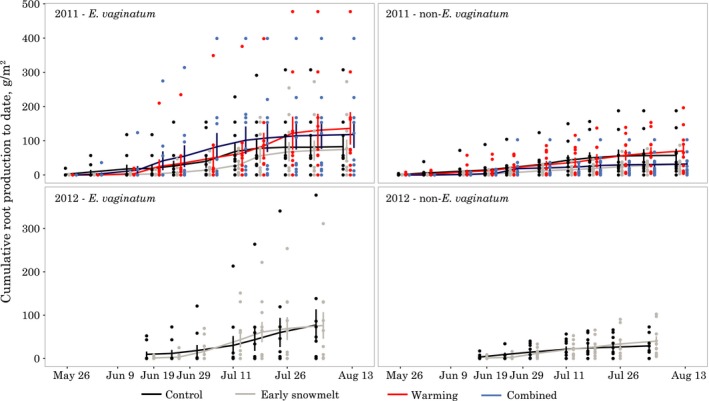
Cumulative root production to date from minirhizotron measurements in 2011 and 2012. Lines and bars show mean ± *SE* by treatment (*n* = 10). Treatments were measured simultaneously but are offset slightly to avoid overplotting. Due to destructive harvests in the open‐top chamber plots, only the control and early snowmelt plots were monitored in 2012

At the end of the 2011 season, cumulative *E. vaginatum* root production in the control plots was 83 ± 29 g/m^2^ (mean ± *SE*, *n* = 10) and 77 ± 36 g/m^2^ in 2012 (Figure 6a). As an example of the range among minirhizotron tubes, in 2011 the 10 control tubes ranged from 0 to 307 g/m^2^ (Figure A6). Non‐*E. vaginatum* roots had lower production than the *E. vaginatum* roots (e.g., 2011 controls = 57 ± 19 g/m^2^; 2012 controls = 29 ± 9 g/m^2^). In 2011, cumulative non‐*E. vaginatum* fine root production in the 10 control tubes ranged from 0 to 188 g/m^2^. In 2011, mean non‐*E. vaginatum* root biomass was lower in early snowmelt and combined treatments than in the control treatment, but the differences were poorly constrained for comparisons of the individual treatments with the control (*n* = 10, early snowmelt non‐*E. vaginatum* *T*_r_ = 0.54 [0.25, 1.27], combined = 0.56 [0.22, 1.45]). However, if the two early snowmelt treatments are pooled together (regardless of warming treatment), there is evidence of a root growth reduction in the early snowmelt treatments for non‐*E. vaginatum* roots characteristic of intertussock microsites during the 2011 season (*n* = 20, *T*_r_ = 0.49 [0.28, 0.89]). This difference was not apparent in the *E. vaginatum* roots (*T_r_* = 0.88 [0.44, 1.76]) or in either root type in 2012 (*T*_r_ = 0.98 [0.29, 3.47] for *E. vaginatum*, 1.38 [0.6, 3.22] for non‐*E. vaginatum*).

### 3.5 Microbial biomass

**Figure 7:**
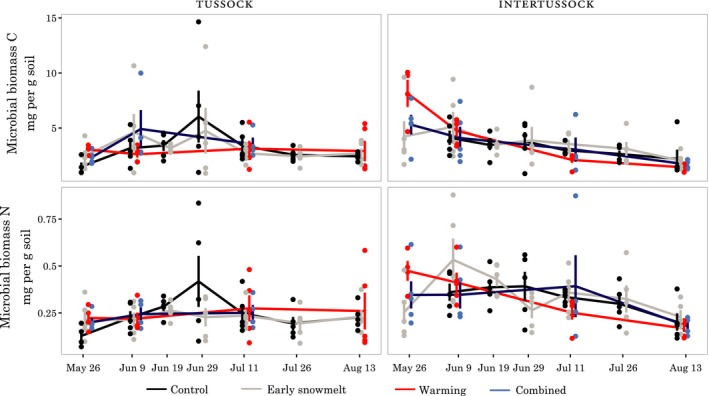
Microbial biomass C (MBC) and microbial biomass N (MBN) measured using the chloroform fumigation technique (mean ± *SE*) during the 2012 season for all four of our treatments (*n* = 5). Treatments were measured simultaneously but are offset slightly to avoid overplotting

During the 2012 season, microbial biomass C (MBC) values in control plots ranged from 1.5 ± 0.3 to 6.0 ± 2.4 mg/g soil (mean ± *SE*) in tussock samples and 2.2 ± 0.8 to 4.1 ± 0.7 mg/g in intertussock samples. On the first measurement date, we observed elevated MBC and MBN in almost all treatments in tussock and intertussock microsites (Figure 7). This elevated microbial biomass effect was most pronounced in the warming‐only treatments (MBC intertussock warming *T*_r_ = 2.2 [1.4, 3.1], MBC tussock warming *T*_r_ = 2.1 [1.2, 3.2], MBN intertussock warming *T*_r_ = 2.0 [1.5, 3.5], MBN tussock warming *T*_r_ = 1.7 [1.2, 2.9]). The exception was intertussock samples in the early snowmelt treatment (e.g., MBC intertussock early snowmelt *T*_r_ = 1.1 [0.6, 2.2]; Figure 7). After this first date, *T*_r_ confidence intervals almost all included 1, and while high variation led to poorly constrained treatment effect sizes, there was no strong evidence of continued difference in MBC or MBN between control and treatment plots.

### 3.6 Microarthropods

**Figure 8:**
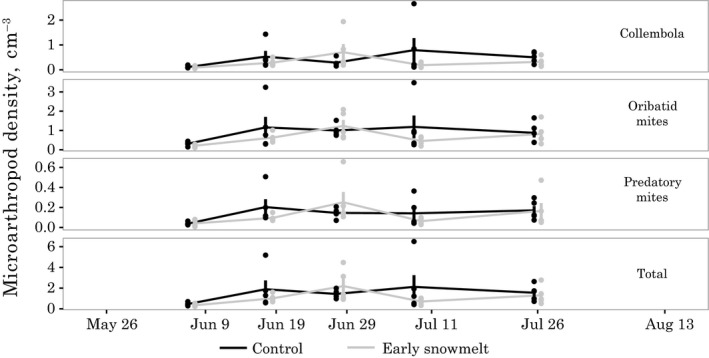
Microarthropod density measured during the 2012 season for early snowmelt and control treatments (*n* = 5). Treatments were measured simultaneously but are offset slightly to avoid overplotting

On the first measurement date, 7 June 2012, total soil microarthropod density, as measured by the sum of the dominant groups of organisms (Collembolans, Oribatid mites, and Predatory mites), was the lowest of the season in both treatments (Figure 8; control = 0.46 ± 0.07 animals/cm^3^; early snowmelt = 0.34 ± 0.06 animals/cm^3^). There was some evidence of a greater magnitude of Oribatid mites in the control plots relative to the early snowmelt plot (*T*_r_ = 0.67 [0.44, 1.03]), which drove the same trend in the overall microarthropod density (early snowmelt *T*_r_ = 0.74 [0.48, 1.09]). On subsequent dates throughout the summer, microarthropod densities varied, but no consistent treatment effect was observed among dates and different microarthropod groups, with mean differences among treatments often driven by single samples with high density. Overall, we saw no evidence of an effect of the early snowmelt treatment on densities of any of the three groups or on total microarthropod density.

### 3.7 Soil pore water nitrogen

**Figure 9:**
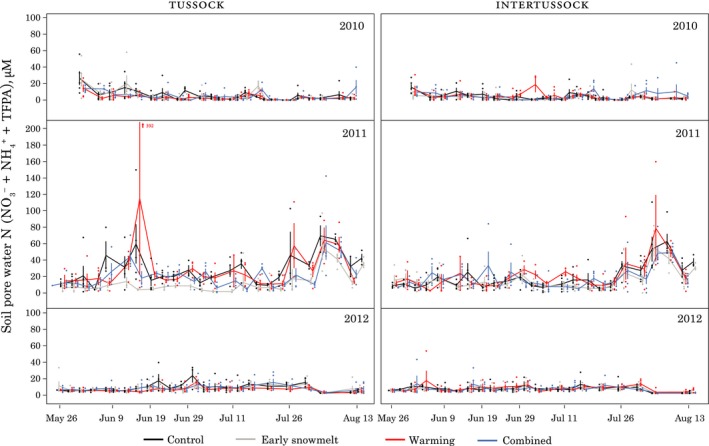
Soil pore water labile N nitrogen (NO_3_^+^ + NH_4_^+^ + TFPA) mean ± *SE* by treatment (*n* = 5). Treatments were measured simultaneously but are offset slightly to avoid overplotting. One outlier (392 µM) is shown by an arrow in the 2011 tussock panel

**Figure A8:**
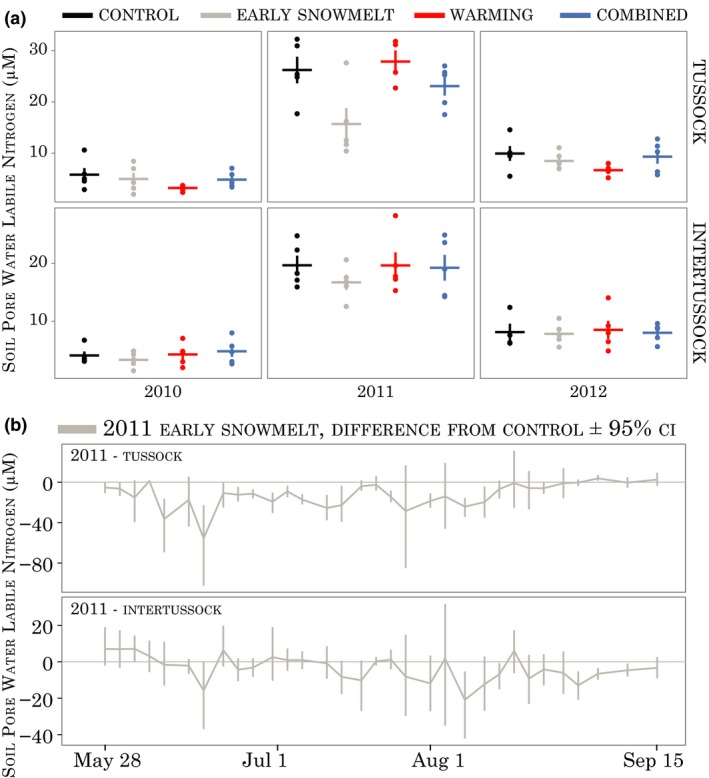
Seasonal averages of labile N. Mean ± SE by treatment (n = 5) are shown as bars, with each point indicating the seasonal average of one microlysimeter measured across 12‐32 dates; b. Analysis of the effects of the early snowmelt treatment in tussock soils throughout the 2011 season for intertussock and tussock soil pore water samples. Difference from control (mean ± bootstrap 95% CI) is shown for each date.

In 2010 and 2012, average seasonal soil pore water labile N (NO_3_^+^ + NH_4_^+^ + TFPA) was low (<10 µM; Figure 9). With low values in all treatments, differences between treatments and controls were likewise low, showing a well‐constrained lack of effect (treatment–control = <5 µM for most treatments in all 3 years). However, in 2011, seasonal averages were higher and available labile N was reduced in the early snowmelt treatment, especially in the tussock soils (2011 early snowmelt tussock soils *T*_r_ = 0.6 [0.41, 0.89]; Figure A8). In examining the specific timing of this effect, lower soil pore water labile N levels were seen in tussock soils beginning on 9 June 2011, and this difference persisted throughout the season until mid‐August, approximately the time of plant senescence. In the intertussock soils, there was some evidence for a smaller effect in that same year driven by dates later in the season (2011 early snowmelt intertussock soils *T*_r_ = 0.85 [0.69, 1.04]; Figure A8).

### 3.8 Soil‐core‐extractable nitrogen

**Figure 10:**
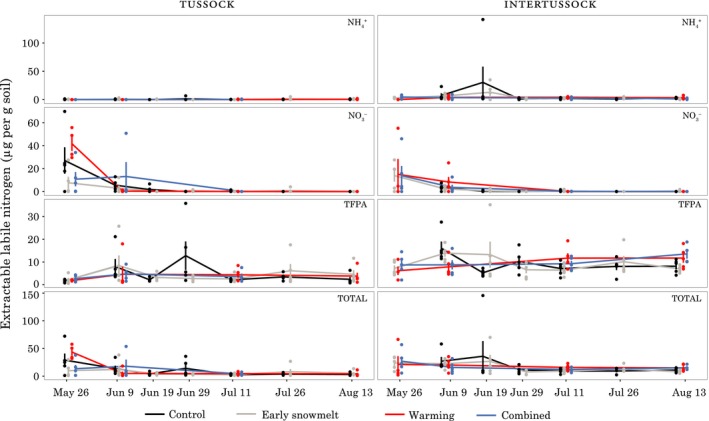
Salt‐extractable NH_4_^+^, NO_3_^−^, TFPA, and their sum (labile N) in intertussock soil cores (left) and *Eriophorum vaginatum* tussock soil cores (right). Note differing scales on the vertical axis. Treatments are offset to avoid overplotting though all treatments were sampled on the same date

Salt‐extractable labile N (NH_4_^+^ + NO_3_^−^ + TFPA) values ranged from 1.01 µg N/g to 145.7 µg/g, with a median of 8.87 µg/g. The values were positively skewed, with the highest 5% of measured values accounting for 24% of the total extractable labile N observed over the 2012 season. The control and warming treatments (i.e., the nonearly snowmelt treatments) in tussock soils showed an early‐season pulse in N driven by nitrate (Figure 10). If we assume no interaction effect in the combined treatment and evaluate the early snowmelt and control treatments regardless of warming treatment, N was reduced in the early snowmelt treatments (early snowmelt = 11.7 ± 4.1, control = 35.5 ± 7.2; *T*_r_ = 3.04 [1.47, 7.88], *n* = 10). This trend was driven by NO_3_^−^ and was not apparent during subsequent dates. The intertussock soils did not show the same trend, instead showing the highest mean value on the third sampling date in 19 June 2012, 35.6 ± 27.4 µg/g in control plots. That peak, however, was driven by a hot spot in a single sample that had a concentration of 146 µg/g dissolved N (142 µg/g of which was NH_4_^+^), our highest observed extractable labile N concentration. Finally, beginning on the fourth measurement date, 11 July 2012, levels of extractable labile N dropped to <15 µg/g in intertussock and <10 µg/g in tussock soil, with little difference between the treatments.

### 3.9 Exoenzyme activities

**Figure 11:**
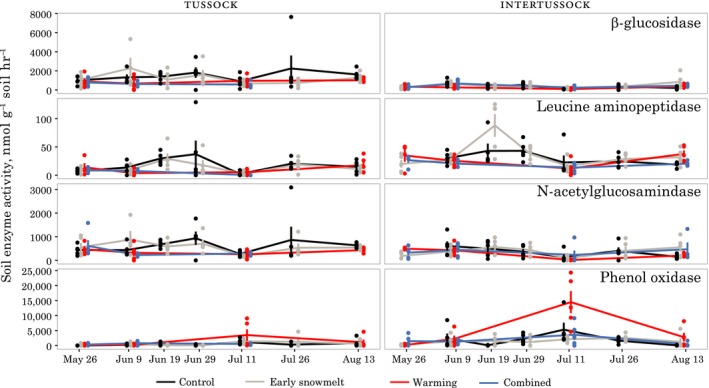
Exoenzyme activities in intertussock soil cores (left) and *Eriophorum vaginatum* tussock soil cores (right). The four enzymes measured are shown in the right panels. Note differing scales. Treatments are offset to avoid overplotting though all treatments were sampled on the same date

For most of the season, measurable activities were present for the hydrolytic enzymes we assayed (medians across all dates and treatments: BG: 548 nmol g^−^^1^ soil hr^−^^1^, NAG: 321 nmol g^−1^ hr^−1^, LAP: 16 nmol g^−1^ hr^−1^). Oxidative enzymes (phenoloxidase), though not directly comparable to the hydrolytic enzyme rates, showed a median rate of ~420 nmol g^−1^ hr^−1^. We observed a peak, particularly in the warming treatment, on one date, 11 July 2012; this occurred at the same time that there was a low point in the hydrolytic enzyme activities (Figure 11). In intertussock soils, on the first measurement date, warming increased both leucine aminopeptidase (LAP) activity (warmed *T*_r_ = 2.8 [1, 8.2], combined *T*_r_ = 2.1 [1.1, 5.5]) and *N*‐acetyl‐α‐d‐glucosaminidase (NAG) activity (warmed *T*_r_ = 3.3 [2.5, 4.7], combined *T*_r_ = 2.2 [0.9, 3.9]). There was less evidence of a treatment effect in the early snowmelt treatment alone, though effect sizes were not well constrained (early snowmelt LAP *T_r_* = 1.6 [0.6, 4.5], early snowmelt NAG *T*_r_ = 1.2 [0.5, 2.3]). Activity rates in tussock samples were more variable, and no obvious treatment effects were observed at any time during the season.

### 3.10 Interannual variation in N cycling

Warmer fall and winter temperatures during our second measurement season (following the 2010–2011 fall and winter; Table 1) were associated with higher labile N, microbial N, and N‐acquiring enzyme activities in soil cores collected at the time of the 2011 thaw compared to those collected in 2010 and 2012 (e.g., 2011–2010 tussock labile N *T*_r_ = 2.34 [1.34, 3.39], MBN *T*_r_ = 2.17 [1.43, 3.1], LAP *T*_r_ = 68.66 [23.67, ∞], NAG *T*_r_ = 2 [1.36, 2.9]). The pattern was not perfect for every variable (e.g., 2012–2011 tussock labile N *T*_r_ = 0.9 [0.32, 1.91]), but overall, the association was supported by the data (Table 1). Maximum snow depth at thaw was observed in 2012, but that was not the year with the warmest overwinter temperatures, which was the winter preceding the 2011 growing season.

## 4 DISCUSSION

Our two hypotheses were (a) early snowmelt advances seasonal timing of soil processes; and (b) early snowmelt damages plants, leading to several possible belowground effects. To assess these hypotheses, we discuss responses of three components of the soil biogeochemical system in our experiment: plants, decomposers, and soil nutrients. In discussing these components, we make the case that there was not strong support for either hypothesis; instead, belowground responses to our early snowmelt treatment were limited in the face of the relatively small changes in soil conditions that our treatments created.

### 4.1 Plant responses

Because no root production data were available in 2010 and the snowmelt advancement period was small (4 days), we begin by discussing plant responses in 2011. We will subsequently return to the evidence of small but detectable changes in aboveground plant phenology in 2010. In 2011, the small advance of aboveground phenology (~1–4 days) in the early snowmelt plots was not commensurate with the length of the snowmelt advancement (15 days). The small magnitude of this effect is in contrast to snow fence manipulations that show strong multiyear directional changes in plant productivity and community composition due to deeper snow (Legault & Cusa, 2015; Wahren, Walker, & Bret‐Harte, 2005; Walker et al., 1999; Wipf & Rixen, 2010). In other words, we observed less dramatic changes in phenology in the opposite direction. This lack of symmetry in effects likely occurs because plants cannot take advantage of early snowmelt due to unfavorable conditions during that time (Bokhorst, Bjerke, Tømmervik, Callaghan, & Phoenix, 2009; Wipf et al., 2009). During the 15‐day snowmelt advancement period in 2011, our snow‐free plots were exposed to very cold air temperatures for ~10 days, suggesting that temperatures during an early snow‐free period determines whether plants can begin to grow earlier. Similar interannual variation in the size of the effect of early snowmelt was observed in a previous multiyear early snowmelt study (Khorsand Rosa et al., 2015). In that study, bud break occurred several days earlier depending on species, but in some years, there appeared to be little difference. Air temperature was cited as a strong predictor of phenology, and this is in line with the interannual differences we see here.

Plant root production showed evidence of reduction in 2011 when we pooled the two warming treatments together, providing some support for our second hypothesis that early snowmelt can negatively affect root growth. This finding is consistent with other snow removal studies in showing that cold temperatures during early snowmelt years can damage plants (Inouye, 2008; Wipf et al., 2006, 2009). However, aboveground plant phenology and NDVI did not show the same type of negative response to early snowmelt (it was weakly positive), which is counter to what we would expect since aboveground tissues are often more sensitive to frost damage than roots (Larcher, Kainmüller, & Wagner, 2010). This lack of aboveground effect suggests that damage to aboveground parts of the plant was not the mechanism behind the reduced root growth. Although we are not sure of the mechanism, one possibility is the fluctuations we observed in soil temperatures. During the 15‐day advancement of snowmelt, the minimum soil temperature did not drop *below* the controls, as was seen in a similar early snowmelt experiment (Wipf et al., 2009), but soils did refreeze and undergo daily temperature cycles. Such daily temperature cycling was not seen in the controls. These freeze–thaw cycles could damage plant roots (Tierney et al., 2001). The root growth reduction effect was only present in non‐*E. vaginatum* roots (possibly because *E. vaginatum *root systems are annual and the plants did not have roots at that time), suggesting that in our system, different species may be more tolerant of exposure to early frost conditions than others.

In 2012, the ecosystem exhibited a markedly different response to our treatments, with a larger advancement of aboveground plant phenology in response to early snowmelt and warming treatments. Unlike in 2011, the NDVI responses were noticeable, and although small in magnitude due to the narrow range created by our measurement approach and calculation as trajectories instead of raw values, the data do suggest a difference in the timing of vegetation greening. These results suggest that in years with warmer early spring temperatures such as 2012, plants can take better advantage of early snowmelt by leafing out earlier (Livensperger et al., 2016). The acceleration of phenology in our OTC treatments is also consistent with other OTC warming experiments that have shown earlier bud break and flowering (Arft et al., 1999; Henry & Molau, 1997). However, despite this much larger aboveground plant response, we did not see evidence for a commensurate effect on root production in either tussock or intertussock roots. Part of the reason may be that root growth peaks later than shoot growth, more toward the middle of the season (Kummerow & Russell, 1980). By the time roots were reaching maximum growth, the effects of the early snowmelt may no longer have had a notable influence.

Given the lack of evidence for a root growth effect in 2012 when there was a much larger advancement in snowmelt, it is unlikely that a large plant root response was present in 2010, either, when we had a smaller advancement in snowmelt (4 days), reflected as small but detectable changes in aboveground plant phenology as measured by the marked individuals. Taken together, our plant responses suggest two main conclusions: (a) For both above and belowground plant components, weather conditions during snowmelt advancement will likely affect how plants respond, with favorable warm conditions potentially accelerating bud break and leaf area development, and cold conditions leading to lack of response or damage; and (b) root growth responses may not mirror aboveground responses in phenology, with roots of at least some species responding in a more limited fashion than aboveground phenology.

### 4.2 Decomposer responses

The early‐season treatment responses in microbial biomass and potential enzyme activities suggest that both warming and early snowmelt (at least in tussocks) caused a small advance of the seasonal microbial succession patterns that have been observed in association with snowmelt in tundra ecosystems (Lipson, Schadt, & Schmidt, 2002; Schmidt et al., 2007). This finding makes sense with the expected physiological effects of a warmer soil environment (Schimel et al., 2004; Wallenstein et al., 2009), but contrasts with one study that suggests the opposite effect (Tan, Wu, Yang, & He, 2014). The earlier onset of isothermal (0°C) conditions in the upper soil in multiple treatment years likely stimulated soil microbial communities as unfrozen water increased in soil (Mikan, Schimel, & Doyle, 2002; Monson et al., 2006). The cycling patterns in soil temperature noted above in the context of the roots may also have affected microbial activity levels and turnover (Yergeau & Kowalchuk, 2008). The reason for the lack of a detectable effect in the intertussock early snowmelt treatment is unclear, but could be linked to the weaker soil temperature response observed in the intertussock iButton temperature data. The disappearance of this early‐season effect by the second measurement date suggests that microbial communities rapidly responded to the convergence of environmental conditions, an effect that could be driven by their rapid generational turnover times even in tundra soils (Schmidt et al., 2007). This small and transient effect is not consistent with either of our hypotheses. Our data instead suggest that while early snowmelt may alter the timing of microbial activities early in the season, these effects will not cascade into season‐long changes to ecosystem function.

Densities of microarthropods were low during the early‐season sampling (7 June) in both the early snowmelt and control treatments, suggesting that earlier snowmelt does not necessarily lead to earlier microarthropod activity, unlike in warming experiments where more substantial responses have been documented (Dollery, Hodkinson, & Jonsdottir, 2006; Hodkinson et al., 1998; Sistla et al., 2013). The lack of treatment response could be because Arctic microarthropods are already well adapted to the freeze–thaw cycles observed in both the control and early snowmelt treatments (Konestabo, Michelsen, & Holmstrup, 2007; Sjursen, Michelsen, & Holmstrup, 2005) and because while these organisms are sensitive to low temperatures after resuming activity in the springtime, soil temperatures were still high enough for activity (Hodkinson et al., 1998). The microarthropods that we sampled are also primarily saprotrophs and thus do not rely directly upon the living plant community (Koltz, Asmus, Gough, Pressler, & Moore, 2018), which may buffer them from some of the resource‐associated effects of early snowmelt. Had we sampled belowground herbivores in 2011 for example, we may have seen greater response due to reductions in root growth of some species. Populations of mites and Collembola increased between our first two measurement dates, 7 June and 18 June. Based on the limited differences in the microbial biomass among treatments after the first measurement date, it is possible that an overall return to control‐like conditions in the early snowmelt plots occurred quickly enough to prevent a measurable impact on these organisms. Overall, these findings suggest that modest changes in the timing of snowmelt alone in the tundra may not have a major effect on the phenology or density of soil‐dwelling microarthropods, particularly on those that emerge well past snowmelt.

### 4.3 Soil nutrient responses

We observed two main treatment effects in soil nutrient dynamics, a transient effect, similar to microbes, in the extractable nutrients in 2012, and a season‐long reduction in soil pore water N in the early snowmelt tussock plots during the 2011 season. While not undetectable or negligible, these effects were situational and do not amount to strong evidence in support of our hypotheses, which would predict larger alterations in the timing of important nutrient cycling events. The OTC warming treatment, more so than the combined treatment, enhanced N cycling at the time of soil thaw, with higher microbial N, labile N, and N‐acquiring enzyme activity. While these differences were not apparent later in the season, they indicate that previous studies in which only a few measurements were taken throughout the summer (Hobbie & Chapin, 1998; Jonasson, Havström, Jensen, & Callaghan, 1993; Kudernatsch, Fischer, Bernhardt‐Romermann, & Abs, 2008) could miss some of these short‐lived effects. The cause of the season‐long reduction in soil pore water nutrients in 2011 is not known. It could be linked to the observed root growth reductions via a mechanism such as rhizodeposition (Cardon & Gage, 2006; Weintraub et al., 2007). However, we saw the nutrient reduction in tussock soils while the root reduction was in non‐*E. vaginatum* roots (though on the other hand, we note that non‐*E. vaginatum* roots such as those from *V. vitis‐idaea* are often present in *E. vaginatum *tussocks). Regardless of the mechanism, because this effect was only apparent in one soil type (tussock) and was only seen in one of 3 years, it does not represent strong evidence that soil nutrient dynamics will be altered in a consistent and predictable fashion by early snowmelt.

Similar to previous studies, we saw evidence of a nutrient crash in the form of low soil‐core‐extractable N concentrations by our third measurement date in 2012 (Weintraub & Schimel, 2005). The soil pore water lysimeter data showed different patterns, with no clear crash. This difference in dynamics was likely due to the substantially different nutrient pool that soil pore water lysimeter measures. The soil cores capture adsorbed and possibly physically protected N in addition to the labile N captured by soil pore water lysimeters that is mobile in the soil pool (Darrouzet‐Nardi & Weintraub, 2014). The crash was more evident in the depletion of this more protected pool, with the more mobile pool staying at relatively low levels for most of the season, consistent with the idea that plants and microbes will draw levels of easily available limiting nutrients down to very low levels (Hobbie & Hobbie, 2012). Despite this observation of nutrient crash dynamics in the soil core extractions, the hypothesis that the timing of the crash would change with our treatments can be rejected. In the soil core extractions, the reduction of extractable N to low levels occurred simultaneously among treatments. Furthermore, we saw no evidence of large timing differences in the soil pore water samples, with seasonal trends among treatments tracking one another well.

### 4.4 Interannual variation

The interannual variations in early‐season labile N, microbial biomass N, and N‐acquiring enzyme activity suggest that warmer overwinter temperatures led to enhanced overwinter N cycling activity that can increase nutrient availability at the time of thaw and throughout the following growing season. The soil pore water N data were particularly striking: following a warmer 2010–2011 snow season, the 2011 soil pore water N concentrations were higher throughout the season. Previous studies have suggested that higher microbial biomass N at the end of winter can affect the total amount of N available to plants at the beginning of the growing season (Buckeridge & Grogan, 2008). Also, overwinter soil enzyme potentials are often highest at the end of winter, just before soil thaw (Wallenstein et al., 2009). The importance of overwinter temperatures in regulating soil processes is supported by the results of snow fence manipulation studies, which have shown that deeper snowpacks can enhance N cycling by warming soils (Borner et al., 2008; DeMarco, Mack, & Bret‐Harte, 2011; Natali et al., 2012; Schimel et al., 2004). While we did not observe an association between snow depth at thaw and overwinter temperatures in 2011 versus 2012, this may be due to differences in air temperatures relative to the timing of snowfall between the 2 years. However, regardless of snow depth at thaw, the associations with warmer overwinter temperatures suggest a causal effect. Microbes can be active at temperatures below 0°C, and thus, even small increases in winter temperatures may have substantial effects on overwinter N mineralization (Mikan et al., 2002; Monson et al., 2006). These interannual differences support our conclusion that our manipulations had less impact on the belowground ecosystem than expected because they did not have a large enough effect on soil temperatures and thaw depth. Large interannual variations are big enough to drive major differences in these belowground processes, but a modest change in snowmelt timing with little concomitant change in soil temperatures for about 2 weeks was not enough.

## 5 CONCLUSION

We did find some effects of our treatments on belowground processes. We were able to document several clear differences among treatments, including the reduced nutrient availability in tussock soils in 2011, reduced root growth in non‐*E. vaginatum* roots that same year, and the clear separation of treatments in soil microbial biomass, enzyme activity, and nutrient cycling on the first measurement date in 2012. However, our documentation of these small effects also made clear, by contrast, the absence of larger and more consistent effects, such as those we had hypothesized and those that we observed between years. Despite not seeing the hypothesized large belowground effects, we have demonstrated aboveground phenology responses based on interannual variation in climate, and highlighted the physical effects that may occur in soils when snow is removed early, including earlier and more dramatic diurnal temperature cycling. For tundra ecosystems, resistance of the belowground environment to aboveground conditions such as earlier snowmelt, and the rapid capability of belowground organisms to respond to new microclimate conditions could provide some buffering against incremental shifts in climate. In the context of global change in Arctic tundra ecosystems, these results suggest that modest changes in the timing of snowmelt that are not accompanied by warmer air temperature will likely have small effects on ecosystem function, particularly below ground.

## AUTHOR CONTRIBUTIONS

MNW (project leader), HS, PFS, and JPS conceived of the project and acquired funding. All authors performed the experiment with foci on particular measurements: HS and CL on aboveground plants, PFS and AS on roots, ADN and MNW on soil nutrients, and AMK on microarthropods. ADN analyzed the data and led manuscript writing, with all authors contributing to the writing.

## Data Availability

The data used for these analyses are available in a permanent archive (ACADIS, 2013). The specific data sets used in this study and the analysis scripts are available as a Figshare archive: https://figshare.com/articles/snowmelt_belowground/7294835.
